# Phosphorylation of HOX11/TLX1 on Threonine-247 during mitosis modulates expression of cyclin B1

**DOI:** 10.1186/1476-4598-9-246

**Published:** 2010-09-16

**Authors:** Edwin Chen, Xiaoyong Huang, Yanzhen Zheng, You-Jun Li, Alden Chesney, Yaacov Ben-David, Eric Yang, Margaret R Hough

**Affiliations:** 1 Institute of Medical Science, University of Toronto, Toronto, Ontario, M5 S 1A8, Canada; 2 Department of Laboratory Medicine & Pathobiology, University of Toronto, Toronto, Ontario M5 S 1A8, Canada; 3 Department of Medical Biophysics, University of Toronto, Toronto, Ontario M5 S 1A8, Canada; 4 Department of Molecular and Cellular Biology, Sunnybrook Health Sciences Centre, Toronto, Ontario, M4N 3M5, Canada

## Abstract

**Background:**

The HOX11/TLX1 (hereafter referred to as HOX11) homeobox gene was originally identified at a t(10;14)(q24;q11) translocation breakpoint, a chromosomal abnormality observed in 5-7% of T cell acute lymphoblastic leukemias (T-ALLs). We previously reported a predisposition to aberrant spindle assembly checkpoint arrest and heightened incidences of chromosome missegregation in HOX11-overexpressing B lymphocytes following exposure to spindle poisons. The purpose of the current study was to evaluate cell cycle specific expression of HOX11.

**Results:**

Cell cycle specific expression studies revealed a phosphorylated form of HOX11 detectable only in the mitotic fraction of cells after treatment with inhibitors to arrest cells at different stages of the cell cycle. Mutational analyses revealed phosphorylation on threonine-247 (Thr247), a conserved amino acid that defines the HOX11 gene family and is integral for the association with DNA binding elements. The effect of HOX11 phosphorylation on its ability to modulate expression of the downstream target, cyclin B1, was tested. A HOX11 mutant in which Thr247 was substituted with glutamic acid (HOX11 T247E), thereby mimicking a constitutively phosphorylated HOX11 isoform, was unable to bind the cyclin B1 promoter or enhance levels of the cyclin B1 protein. Expression of the wildtype HOX11 was associated with accelerated progression through the G2/M phase of the cell cycle, impaired synchronization in prometaphase and reduced apoptosis whereas expression of the HOX11 T247E mutant restored cell cycle kinetics, the spindle checkpoint and apoptosis.

**Conclusions:**

Our results demonstrate that the transcriptional activity of HOX11 is regulated by phosphorylation of Thr247 in a cell cycle-specific manner and that this phosphorylation modulates the expression of the target gene, cyclin B1. Since it is likely that Thr247 phosphorylation regulates DNA binding activity to multiple HOX11 target sequences, it is conceivable that phosphorylation functions to regulate the expression of HOX11 target genes involved in the control of the mitotic spindle checkpoint.

## Background

Dysregulated expression of homeobox genes is recognized as a common mechanism in the pathogenesis of leukemias [1]. Given their critical roles as transcription factors capable of controlling cellular proliferation and differentiation during embryogenesis, there has been increasing interest in the possible role of homeobox genes as potential proto-oncogenes when their regulation goes awry. One such well-documented instance of homeobox genes possessing oncogenic activity following perturbation of their expression is the HOX11/TLX gene family. To date, three family members of the HOX11/TLX gene family are known: HOX11/TLX1, HOX11L1/TLX2 and HOX11L2/TLX3. HOX11/TLX1 (hereafter referred to as HOX11) and HOX11L2/TLX3 have been reported to be frequent targets of aberrant activation by chromosomal rearrangements in the pathogenesis of T-lineage leukemias. In particular, the HOX11 gene is rearranged in T-cell acute lymphoblastic leukemias (T-ALLs) by recurrent t(10;14)(q24;q11) or t(7;10)(q35;q24) chromosomal translocations [2-4]. The juxtaposition of the HOX11 gene downstream of either the TCRα/δ or TCRβ regulatory elements results in aberrant expression of the homeobox gene in T lymphocytes, a cell type in which it is not typically expressed, leading to T lymphocyte transformation and ultimately, the development of T-ALL. Dysregulated HOX11 expression can also occur in the absence of chromosome translocations with aberrant expression being reported in 3-5% of pediatric and up to 30% of adult T-ALL cases [5-9]. Expression profiling of primary leukemic lymphoblasts from HOX11^+ ^T-ALL patients was indicative of leukemic arrest at an early cortical thymocyte stage of T cell development [7], consistent with immunophenotyping studies, which revealed primary HOX11^+ ^T-ALL samples are predominantly TCRαβ^+ ^and TCRγδ^- ^[10]. Dysregulated HOX11 expression has been reported in a subgroup of T-lymphoblastic lymphomas patients with favourable outcomes [11]. Additionally, retroviral transduction of fetal liver precursors with HOX11 induced maturation arrest prior to the CD4^+ ^CD8^+ ^double positive stage in fetal thymic organ cultures [12]. The transforming capacity of HOX11 overexpression has also been verified in several in vitro and in vivo studies. Overexpression of HOX11 by retroviral transduction was able to immortalize murine hematopoietic and embryonic precursors, transform murine bone marrow cells and lead to the de-differentiation of the murine erythroleukemic cell line J2E in vitro [13-15]. Additionally, we developed a transgenic mouse strain overexpressing HOX11 throughout B cell development that developed spontaneous mature B cell lymphomas with an extended latency [16,17].

The molecular mechanisms of action of *HOX11 *in inducing a tumorigenic state remain unclear, although it is thought that the transactivation of specific downstream genes by *HOX11 *is responsible, at least in part, for cellular transformation. One-hybrid experiments have elucidated three regions of the HOX11 oncoprotein which are important for optimal transactivation of reporter constructs: an N-terminal glycine/proline rich region, a C-terminal glutamine-rich region, and the homeodomain [18]. A threonine residue at amino acid position 247 (Thr247) (also referred to as position 47 within the homeodomain [19,20]), as opposed to the valine or isoleucine residues typically encoded for in the canonical homeodomains of other homeobox genes, is a primary feature of all members of the *HOX11/TLX *gene family [2]. In particular, this residue is thought to participate in modulating the DNA-binding specificity of HOX11. Instead of the canonical TAAT binding site recognized by prototypical class I HOX proteins, the threonine has been reported to impart a preferential association with guanine nucleotides, altering the *HOX11 *consensus recognition motif to TAAGTG [20]. One gene known to be targeted in a Thr247-dependent fashion is aldehyde dehydrogenase 1 (*Aldh1*) [19,21].

A comprehensive structure-function analysis of the HOX11 protein revealed that the transcriptional transactivation of genes specified by the Thr247 residue of HOX11 was distinct from its immortalizing function, as assayed by the ability to immortalize hematopoietic precursors and generate IL3-dependent myeloid-like cell lines [19]. Mutation of the Thr247 residue to the canonical amino acid, isoleucine, failed to abolish HOX11 transforming activity. Conversely, the PBX-interacting motif (PIM), a domain involved in facilitating HOX11 interaction with its cognate PBX cofactors, was required for immortalization. The PIM domain had previously been shown to be unimportant in transcriptional transactivation of *Aldh1 *[22], but was essential in conferring altered DNA-binding specificity of HOX11 to promoters containing PBX-responsive sequences [23]. Thus, this suggests a dual specificity of HOX11 target genes: those dictated by the Thr247 residue within the homeodomain and those dictated by HOX11 interaction with PBX1, with the latter playing an important role in cellular immortalization.

Several reports have implicated HOX11 in disruptions of various cell cycle checkpoints. In Jurkat T cells, *HOX11 *overexpression conferred an ability to aberrantly bypass G_2_/M cell cycle checkpoint arrest induced by gamma-irradiation [24]. High throughput analyses have revealed that Jurkat T cells engineered to express *HOX11 *exhibit aberrant expression of genes involved in the regulation of the G_1_/S cell cycle checkpoint, including E2F, c-Myc, and the cAMP-responsive element binding protein (CREBP) [25]. An independent microarray study performed on T-ALL cell lines overexpressing *HOX11 *demonstrated elevated expression of the cell cycle checkpoint regulators, NFKB2 and SMARCD3 [26]. Our and other groups have previously reported the ability of *HOX11 *to promote bypass of mitotic checkpoint arrest, leading to chromosome missegregation and aneuploidy [27-29]. This abnormal mitotic checkpoint regulation was correlated with abnormal expression of several mitotic checkpoint regulators, including cyclin B1. Collectively, these studies implicate disruption of the cell cycle as an important biological effect of *HOX11 *overexpression, and may represent a critical mechanism by which *HOX11 *elicits lymphoma.

Given the multiple defects in cell cycle regulation subsequent to HOX11 overexpression in several different cellular models, we investigated the expression of the HOX11 protein at various stages of the cell cycle. Surprisingly, we detected a hyperphosphorylation of the HOX11 oncoprotein on the Thr247 residue, which was specific to mitotic cells. The implications of this post-translational modification with respects to cyclin B1 transactivation and mitotic spindle checkpoint were explored.

## Methods

### Plasmid construction

FLAG-tagged wildtype and truncated HOX11 proteins were generated by subcloning polymerase chain reaction (PCR)-amplified fragments, corresponding to the following HOX11 regions in frame with the sequence encoding a FLAG peptide into the HindIII/NotI restriction sites of the p3XFLAG-CMV-7.1 vector (Invitrogen, Burlington, ON): HOX11Δ260 (amino acids 1-260), HOX11Δ187 (amino acids 1-187) and HOX11Δ106 (amino acids 1-106). HOX11 Thr247Ala, Thr247Glu and Thr241Ala were generated using the QuikChange Site-Directed Mutagenesis Kit (Stratagene, La Jolla, CA) prior to cloning as described above. Luciferase reporter constructs were generated by subcloning PCR-amplified fragments corresponding to the following promoter regions upstream of the luciferase reporter gene into the KpnI/HindIII restriction site of the pGL3 vector (Promega, Madison, WI): CCNB1L-LUC (contains -4902 to +21 relative to TSS of murine cyclin B1 (CCNB1) gene) and CCNB1S-LUC (contains -3310 to +21 relative to TSS of murine cyclin B1 (CCNB1) gene). All constructs were verified by DNA sequencing.

### Materials and cell lines

NIH 3T3 fibroblasts stably expressing the HOX11 oncoprotein were generated by transfection of NIH 3T3 cultures with 3 μg MSCV-HOX11 plasmid constructs using the Polyfect Transfection reagent (QIAGEN), and selecting with 800 μg/ml G418 (Sigma) for 14 days. Media changes supplemented with fresh G418 were performed every two days. Following 14 days of selection, 24 G418-resistant colonies were picked, expanded and tested for HOX11 positivity by Western immunoblotting with a HOX11-specific antibody (Santa Cruz Biotechnology, Santa Cruz, CA). HOX11-3T3 fibroblasts were maintained in Dulbecco's Modified Eagle's Medium (DMEM) supplemented with 10% FCS, 2 mM L-glutamine and 1% penicillin/streptomycin. The T cell leukemia cell lines Jurkat [30] and ALL-SIL [3] were maintained in RPMI supplemented with 20% FCS, 2 mM L-glutamine and 1% penicillin/streptomycin and maintained at a cell density of 0.5-1.5 × 10^6 ^cells/ml. Relative levels of the HOX11 protein in the different cell lines is shown in Additional file 1, Figure S1.

### Phosphorylation experiments

Nocodazole treatment was performed 24 hours after seeding, on typically ~60-80% confluent cultures. Phosphoenriched lysates following nocodazole treatment were generated using the PhosphoCruz Protein Purification System (Santa Cruz) according to manufacturer's protocols. Staurosporine (Sigma) was added in conjunction with nocodazole at concentrations of 0, 20, 40, 60, 80 and 100 nM. A dose-response experiment using staurosporine concentrations from 0.05 μM to 500 μM indicated drug concentrations did not impact cell viability (Additional file 2, Figure S2). Recombinant protein phosphatase 1 (PP1) catalytic subunit (Sigma) was added in conjunction with nocodazole at concentrations of 1, 2 and 4 U/mL.

### Co-immunoprecipitation and immunoblotting analysis

HOX11 co-immunoprecipitations were performed using the Immunoprecipitation Kit with 1 μg anti-HOX11 monoclonal antibody, clone 1D7 (Santa Cruz) according to manufacturer's protocols. Immunoblotting analysis was performed on SDS-separated proteins blotted to nitrocellulose membrane, and probed at various dilutions with a primary antibody recognizing HOX11 (1:2,000), FLAG (1:3,000), pSer (1:100), pThr (1:100), PP1 (1:800), PP2A (1:2,000) and β-actin (1:2,000) for 4 h at room temperature, followed by three washes with PBST for 20 min each. Membranes were subsequently incubated with either anti-mouse (1:10,000) or anti-rabbit (1:10,000) antibody conjugated to horseradish peroxidase (HRP) for 1 h at room temperature. The anti-pSer and anti-pThr antibodies were obtained from Anaspec, the anti-FLAG antibody was obtained from Sigma. All other antibodies were obtained from Santa Cruz Biotechnologies (Santa Cruz, CA).

### Chromatin immunoprecipitation (ChIP)

Chromatin immunoprecipitations were performed using the Chromatin Immunoprecipitation Kit (Upstate) and a HOX11-specific antibody, clone 1D7, (Santa Cruz Biotechnology, Santa Cruz, CA) according to manufacturer's protocols. Primers used for ChIP PCR analyses were:

CYCB1-1F: ACTTCATGCTATCAACCTCA; CYCB1-1R: CCTCTTCTATAGAAGTGCCA;

CYCB1-2F: CCGCGTTAGTGTTACTGAAA; CYCB1-2R: CAAAGATCTCTATTGCAACTCT; CYCB1-3F: TGTAACCTCAACAATGAGGA; CYCB1-3R: ACACCATCCTGTGCTCTCTA;

CYCB1-4F: CAGTCTCCGGTGTGACATAA; CYCB1-4R: GAGACAGGGTTTCTCTGTGT;

CYCB1-5F: AACAAGTTCCAGCCTCCACA; CYCB1-5R: CCCATTACCAAGCTAGAGAG;

CYCB1-6F: AGGAACAGCCAGAGCTGTTT; CYCB1-6R: AAATGCCAATGGTCTCCCTG.

### Luciferase assays

NIH 3T3 fibroblasts were seeded in 24-well dishes 24 hours prior to transfection. Cells were transfected with 0.15 μg FLAG-HOX11 construct and 0.15 μg promoter-reporter construct using the Polyfect transfection reagent (QIAGEN). Each transfection was performed in triplicate. Luciferase assays were performed using the Luciferase Reagent Kit (Promega), according to manufacturer's protocols. Briefly, cells were lysed by addition of 200 μL CCNR lysis buffer, cleared by centrifugation and 20 μL protein lysate was combined with 100 μL luciferase reagent immediately prior to measurement in the luminometer. For each sample, arbitrary luciferase units were standardized to protein concentrations.

### Electrophoretic mobility shift assay (EMSA) analysis

Nuclear extracts were derived from Jurkat cell lines stably expressing an empty Flag vector, Flag-HOX11-wt, Flag-HOX11-T247A or Flag-HOX11-T247E. The oligonucleotide probes derived from the CCNB1 promoter and mutant probe were as follows: 5'-TCCATCCCAGTAATAAGTGTTTT-3' and 5'-TCCATCCCAGTAAGGGCCCTTTT-3'. Double-stranded oligonucleotide probes were generated by annealing equimolar quantities of complementary oligonucleotide in 1× annealing buffer (10 mM Tris-HCl, pH7.4, 50 mM NaCl, 1 mM EDTA). Probes were incubated in 1× binding buffer (10 mM Tris-HCl, pH7.6, 50 mM NaCl, 1 mM EDTA, 5%Glycerol) with 6 μg nuclear extract in a final volume of 10 μl.The samples were incubated at room temperature for 30 min and resolved by electrophoresis in 6% polyacrylamide nondenaturing gels in 0.5 × TBE at 100 voltage for 30 min. Following electrophoresis, the gels were stained using an EMSA staining kit (Invitrogen,Carlsbad,CA,USA) and photographed using a 300 nm UV transilluminator.

### Cell cycle analysis

Cells were synchronized at the G1/S checkpoint by culturing for 16 h in 100 μg/ml aphidocolin, released into S phase by feeding with fresh medium containing 10% fetal calf serum for 4 h, arrested at G2/M by culturing with 60 μg/ml genistein for 16 h, or trapped in mitosis by using 400 μg/ml colchicine, 50 nM or 100 nM nocodazole (for ~50% accumulation or maximal accumulation of cells in metaphase, respectively) or 3 μg/ml cytochalasin B (for cytokinesis arrest following telophase) for 16 h. Cell cycle distribution was determined by propidium iodide staining and flow cytometry. For each sample, 20,000 events were recorded.

### Mitotic index

Cells were co-transfected with a pmax GFP-expressing vector (Amaxa) and either an empty vector, a vector expressing a wildtype HOX11 or a vector expressing the HOX11Thr247Glu mutant. After 24 h, cells were synchronized in mitosis by a 17 h exposure to colchicine (40 ng/ml) or (10 ng/ml) and stained with Hoechst 33342 (10 μM) in 2% FCS/PBS in 37°C for 10 min. Mitotic indexes of transfected cells were quantified using immunofluorescent microscopy by scoring the nuclear morphology of 200 GFP-expressing cells, with bright-stained nuclei corresponding to prometaphase arrested cells and dull or diffuse-stained nuclei corresponding to cells that had escaped mitotic arrest. Cells were viewed with an Axiovert 200 M epi-fluorescence microscope using Axiovision Rel. 4.5 software (Carl Zeiss, Toronto, ON, Canada). Each experiment was replicated three times.

### Apoptosis assays

Cell death was monitored by flow cytometric analysis of cells stained by Annexin V (Biovision ) and propidium iodine (PI) (Invitrogen), as per the manufacturer's protocol. 30,000 cells were analyzed.

## Results

### HOX11 is phosphorylated during mitosis

**Figure 1:**
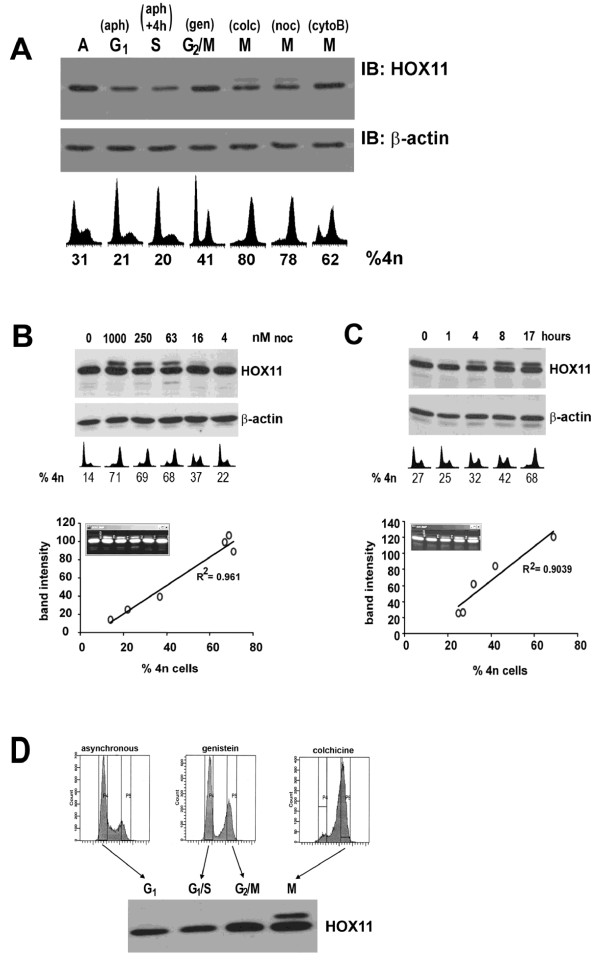
**HOX11 is Post-Translationally Modified During Mitosis in a Dose- and Time-Dependent Manner in Proportion to the Percentage of Mitotic Cells**. **(A) **Immunoblot analysis of HOX11 and β-actin levels in HOX11-3T3 cultures following treatment with 100 μg/ml aphidocolin (aph), aphidocolin followed by release into fresh medium containing 10% fetal calf serum for 4 h (aph+4h), 60 μg/ml genistein (gen), 400 μg/ml colchicine (colc), 100 nM nocodazole (noc) or 3 μg/ml cytochalasin B (cytoB). Flow cytometric profiles of DNA content and percentage of cells containing a 4n complement of DNA are indicated. **(B) **Immunoblot analysis of HOX11 and β-actin expression in HOX11-3T3 cultures treated with varying doses of nocodazole for 17 hours **(C) **or with 50 nM nocodazole for varying times. Flow cytometric profiles of DNA content and percentage of cells containing a 4n complement of DNA are indicated. Densitometric analysis of post-translationally modified band intensity compared to the percentage of cells containing a 4n complement of DNA reveals a statistically significant linear relationship (R^2 ^> 0.9). **(D) **Immunoblot analysis of HOX11 expression in sorted HOX11-3T3 subpopulations following Hoechst 33342 staining.

To assess HOX11 expression levels at various stages of the cell cycle, fibroblast cell lines stably expressing the HOX11 oncoprotein (HOX11-3T3) were treated with various inhibitors to induce cell cycle arrest at different stages. HOX11-3T3 cells were synchronized at the G1/S checkpoint by culturing for 16 h in aphidocolin and released into S phase by feeding with fresh medium for 4 h, arrested at G2/M by culturing with genistein for 16 h, blocked in mitosis by exposure to colchicine or nocodazole for 16 h or arrested during cytokinesis following telophase with cytochalasin B for 16 h. While no dramatic cell cycle-related fluctuations in HOX11 expression levels were detected by immunoblotting, we observed the presence of a gel retarded cross-reacting band when HOX11-3T3 cultures were treated with colchicine or nocodazole (Figure 1A), but not following treatment with the G2/M inhibitor, genistein or the cytokinesis inhibitor, cytochalasin B. We hypothesized that the additional band represented a post-translationally modified version of the HOX11 peptide.

The lower mobility band was detected in a nocodazole dose-dependent manner (Figure 1B) and in a time-dependent manner (Figure 1C). Densitometry verified that the presence of the higher molecular weight band exhibited a linear correlation with the percentage of cells arrested in the G2/M phase of the cell cycle comprising a 4n complement of DNA, suggesting that the post-translational modification was specific to cells in G2- or M-phase. To verify this, viable cell sorts of Hoechst 33342 stained HOX11-3T3 cells were performed using flow cytometry on asynchronous, genistein and colchicine treated cultures, and subjected to immunoblot analysis (Figure 1D). Asynchronous cultures and cultures in G1 (lanes 1 and 2, respectively) did not exhibit a HOX11 post-translational modification. Similarly, cells arrested at the G2/M boundary using genistein (lane 3) also did not exhibit the HOX11 post-translational modification. As genistein induces arrest prior to entry into M-phase, this suggested that the presence of a 4n complement of DNA following DNA replication alone was insufficient for the induction of the cross-reacting band. Rather, only cells arrested in mitosis (lane 4) following colchicine treatment exhibited the higher molecular weight HOX11 peptide. Attempts to perform HOX11 immunoblot for non-4n fractions of these colchicine-treated cultures to eliminate the possibility of a direct colchicine effect on the cultures were attempted but failed owing to the limited number of non-mitotic cells in these cultures. However, we believe the effect was unlikely to be a direct effect of colchicine, as the same phenomenon was seen with another mitosis-arresting drug, nocodazole. Furthermore, the effect was not likely to be a general effect of genomic instability caused by spindle poisons, as treatment with ethidium bromide and acrylamide, agents capable of inducing genomic instability but not mitotic arrest, had no effect on eliciting the cross-reacting band (data not shown). Collectively, these data provide evidence that the post-translational change was specific to the mitotic fraction. Interestingly, the unmodified HOX11 protein was still readily detectable in this cell fraction. This may be attributable to the cellular heterogeneity within this fraction of cells which has been shown, using immunostains for phosphohistone mitotic markers, to comprise both mitotic cells and post-replicative, non-mitotic (G2) cells [31]. Alternatively, this may reflect an incomplete mechanism for post-translational modification of the HOX11 peptide.

**Figure 2:**
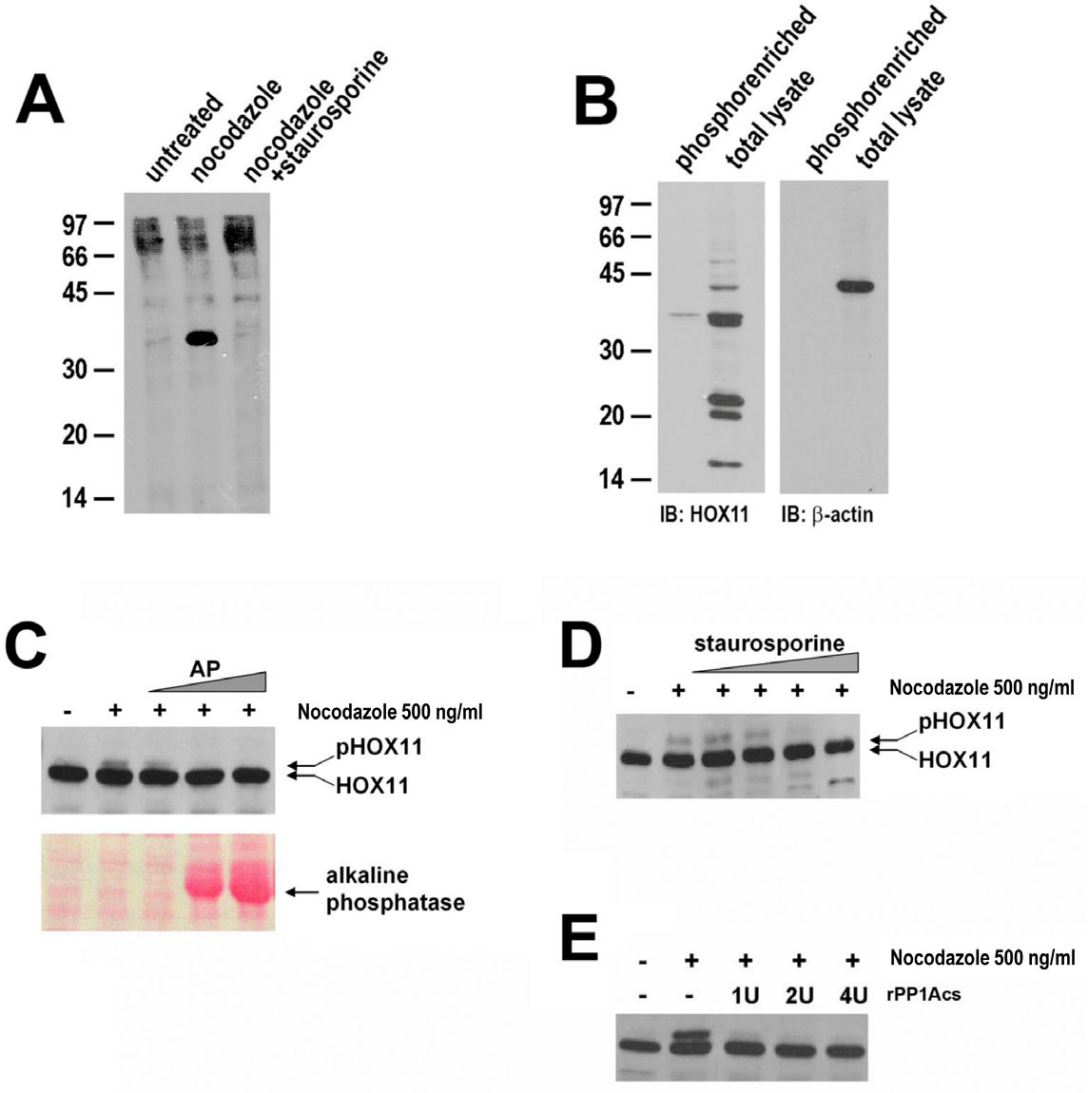
**HOX11 is Phosphorylated During Mitosis**. **(A) **HOX11 immunoprecipitates from 35S-γATP radiolabelled HOX11-3T3 cultures following treatment with nocodazole (500 ng/ml) or nocodazole supplemented with staurosporine (50 μM). **(B) **Immunoblots of HOX11 and β-actin protein levels in total and phosphoenriched fraction of nocodazole-treated lysates. **(C) **Immunoblot of HOX11 protein levels of untreated and nocodazole-treated HOX11 lysates following incubation with various levels of alkaline phosphatase. Ponceau S staining demonstrating exogenous addition of alkaline phosphatase to the *in vitro *enzymatic reaction. **(D) **Immunoblot of HOX11 protein levels following treatment with nocodazole and 0.05, 0.5, 5.0 or 50 μM staurosporine. **(E) **Immunoblot of HOX11 protein levels following treatment with nocodazole in media supplemented with 0-4 U of recombinant protein phosphatase 1 catalytic subunit (rPP1cs).

To test if this band represented a phosphorylated form of HOX11, several assays were performed. First, immunoprecipitation was performed on HOX11-3T3 cultures radiolabelled with 35S-γATP. We observed the presence of a radiolabelled HOX11 isoform following induction of mitotic arrest by nocodazole treatment, which was abolished when co-treated with the non-specific kinase inhibitor, staurosporine (Figure 2A). Secondly, lysates were obtained from mitotically arrested cells, enriched for phosphopeptides by immobilized metal affinity chromatography and subjected to HOX11 immunoblot. While a doublet could be detected in non-enriched lysates, only the higher molecular weight, lower mobility band was detected in the phosphoenriched lysates (Figure 2B). Thirdly, total protein lysates were extracted from nocodazole-treated HOX11-3T3 cells by repeated freeze-thaw and subsequently incubated *in vitro *with increasing amounts of alkaline phosphatase for 1 h at 37°C. Western immunoblotting of the entire reaction confirmed the disappearance of the upper HOX11 band upon alkaline phosphatase treatment (Figure 2C). Fourthly, simultaneous treatment of HOX11-3T3 cultures with a non-specific kinase inhibitor staurosporine, in conjunction with nocodazole, abolished the modified form of HOX11 in a dose-dependent manner (Figure 2D). And finally, given previous reports of direct interactions of HOX11 with the catalytic subunits of protein phosphatases PP1 and PP2A [24], we sought to explore whether these protein phosphatases could play a role in regulating the phosphorylated state of HOX11. Indeed, we observed that recombinant PP1 catalytic subunit (rPP1cs), when added in combination with nocodazole to HOX11-3T3 cultures, very efficiently abrogated the upper HOX11 band (Figure 2E). Collectively, this evidence indicates that the lower mobility band represents a phosphorylated form of HOX11.

### HOX11 is phosphorylated on Threonine-247

**Figure 3:**
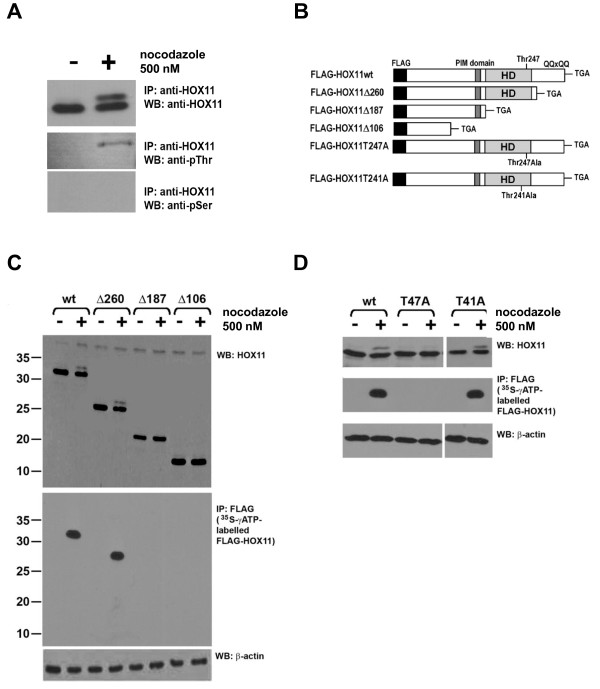
**HOX11 is Phosphorylated on Threonine-247**. **(A) **Immunoblots of anti-HOX11 immunoprecipitates using anti-HOX11, anti-pThr and anti-pSer antibodies. **(B) **Schematic diagram of expression constructs for FLAG-tagged HOX11 wildtype and mutant proteins. FLAG peptide, PBX1-interacting domain (PIM domain), glutamine-rich stretch (QQxQQ) and homeodomain (HD) are noted. **(C) **Top Panel: HOX11 immunoblots of wildtype FLAG-HOX11 and truncation mutants with and without nocodazole treatment. Middle Panel: Autoradiogram of *in vitro *^35^S-γATP-labelled FLAG-HOX11 species. NIH 3T3 cells transfected with wildtype FLAG-HOX11 or truncation mutations were pulsed with ^35^S-γATP either with or without nocodazole. Anti-FLAG immunoprecipitates were obtained as described in the Materials and Methods. Bottom Panel: β-actin immunoblot of wildtype FLAG-HOX11 and truncation mutants with and without nocodazole treatment. **(D) **Top Panel: HOX11 immunoblots of wildtype FLAG-HOX11, T247A and T241A point mutants with and without nocodazole treatment. Middle Panel: Autoradiogram of *in vitro *^35^S-γATP-labelled FLAG-HOX11 species. Bottom Panel: β-actin immunoblot of wildtype FLAG-HOX11 and point mutants with and without nocodazole treatment.

HOX11 immunoblots, performed on anti-HOX11 immunoprecipitates of nocodazole-treated cultures, were able to detect both the non-phosphorylated and phosphorylated HOX11 forms (Figure 3A). To ensure specificity, two different HOX11 antibodies were used for these experiments: a mouse monoclonal antibody directed against the C-terminal domain of HOX11 was used for the immunoprecipitation, whereas a rabbit polyclonal antibody was used for immunoblotting. Additionally, to determine which amino acid residues of HOX11 were phosphorylated, immunoblots were performed using anti-serine and anti-threonine. Only the anti-threonine antibody yielded a signal at an appropriate molecular weight corresponding to that of HOX11 following mitotic arrest (Figure 3A).

To identify the specific residue that was phosphorylated, several FLAG-tagged HOX11 mutants were generated (Figure 3B), and the ability of each mutant HOX11 protein to be phosphorylated was assessed. We observed that a HOX11 mutant protein truncated at amino acid 260 (HOX11Δ260) was capable of being phosphorylated, whereas a mutant protein truncated at amino acid 187 (HOXΔ187) was not, suggesting that the phosphorylated residue likely resided between amino acids 187-260 (Figure 3C). Located within this region is the Thr247 residue which is the defining feature of the *HOX11 *gene family and is known to be integral to the association of DNA binding elements containing the TAAGTG nucleotides. Given its critical role in directing DNA binding, we speculated that it would be a candidate residue for phosphorylation and subsequent modulation of transcriptional activity. To that end, we generated HOX11 point mutants in which the Thr247 residue as well as an adjacent Thr241 residue were mutated to alanine, and assessed their ability to be phosphorylated. We observed that mutation of the threonine residue at amino acid 247 to an alanine (T247A) diminished HOX11 phosphorylation following nocodazole treatment, indicating that HOX11 was being phosphorylated on threonine-247 (Figure 3D). Disruption of an adjacent threonine on residue 241 did not have a similar effect.

### Phosphorylation of HOX11 inhibits cyclin B1 transactivation

We next sought to determine whether the phosphorylation of HOX11 on the Thr247 residue played a role in regulating expression of HOX11 downstream targets. To accomplish this, we assessed the role of Thr247 phosphorylation on modulating the expression of a putative HOX11 downstream target gene, cyclin B1 [29].

**Figure 4:**
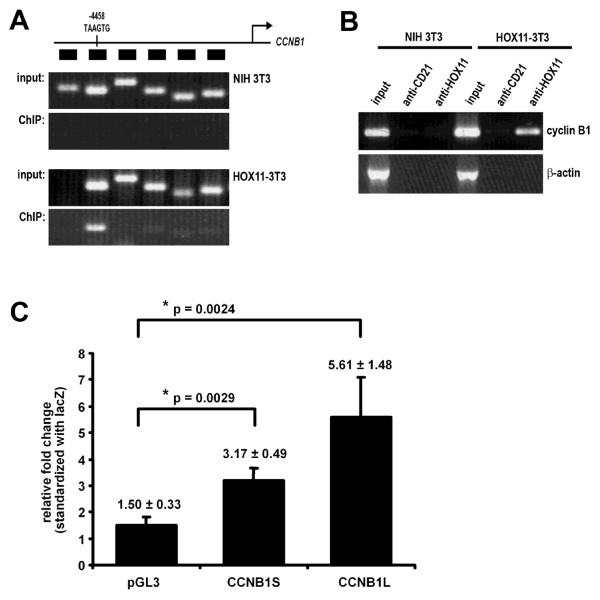
**HOX11 Binds to and Transcriptionally Modulates Expression of the Cyclin B1 (*CCNB1) *Promoter**. **(A) **Chromatin immunoprecipitation (ChIP) analysis using an anti-HOX11 antibody of six regions of the *CCNB1 *promoter region. **(B) **ChIP analysis of region 5 of the *CCNB1 *promoter using an anti-HOX11 antibody and an irrelevant antibody control. **(C) **Promoter-reporter assays to determine HOX11 transactivation of the empty reporter vector (pGL3), the *CCNB1 *promoter containing the TAAGTG consensus site (CCNB1L-LUC, Long) and the *CCNB1 *promoter missing the TAAGTG consensus site (CCNB1S-LUC, Short). Each bar represents the fold ratio of luciferase activity between HOX11- and vector alone-transfected cultures for each promoter-reporter construct. The results are representative of three independent experiments.

We first characterized the promoter region of the cyclin B1 gene (*CCNB1*) with respect to potential HOX11 binding sites. Chromatin immunoprecipitations (ChIPs) were performed to detect direct HOX11 protein association with putative promoter regions up to 5 kb upstream of the transcription start site (TSS) of the *CCNB1 *locus. Six different regions at intervals of 700-1000 bp upstream of the TSS were analyzed. Whereas there was minimal amplification of any of the six regions in NIH 3T3 cells not expressing HOX11, we detected HOX11-binding to four of six regions upstream of the *CCNB1 *gene, with the strongest spanning a region of DNA approximately -4500 to -4300 from the TSS (Figure 4A). This region contains the TAAGTG consensus site proposed by Tang and Breitman (1995) to represent the HOX11 consensus binding sequence. HOX11 association to this region was verified more rigorously by performing ChIP using an irrelevant anti-CD21 isotype-matched control antibody. We were able to reproduce the HOX11 association with the same region of the *CCNB1 *promoter region, which was not seen in cells lacking HOX11 or when we used the anti-CD21 antibody (Figure 4B). EMSA analyses further supported binding of the HOX11 protein to the TAAGTG target sequence (Additional file 3, Figure S3).

In addition to association with the promoter, we assessed the ability of HOX11 to transcriptionally transactivate the *CCNB1 *promoter using luciferase reporter assays. *CCNB1 *promoter-luciferase reporter constructs were generated which encompassed the putative HOX11 binding sequence, TAAGTG, in addition to one which lacked the consensus sequence, designated CCNB1L-LUC (Long) and CCNB1S-LUC (Short), respectively. NIH3T3 cells were cotransfected with a wildtype HOX11 cDNA and either an empty reporter construct or constructs containing CCNB1S-LUC or CCNB1L-LUC and luciferase activity was measured following 24 hours.

Transfection of the empty vector in the absence of HOX11 showed background levels of luciferase activity whereas cotransfection of the CCNB1S-LUC reporter construct with a vector expressing the wild type HOX11 cDNA resulted in a three-fold increase in luciferase activity (3.17-fold ± 0.49, p = 0.0029), demonstrating that the CCNB1 regulatory elements in the presence of HOX11 were active in NIH 3T3 cells. Cotransfection of the CCNB1L-LUC construct with wild type HOX11 significantly enhanced luciferase activity (5.61-fold ± 1.48, p = 0.0024) (Figure 4C). Thus, HOX11 was able to modulate the levels of cyclin B1 expression through interaction with a putative HOX11 binding site situated at approximately -4.5 kb upstream of the *CCNB1 *TSS.

**Figure 5:**
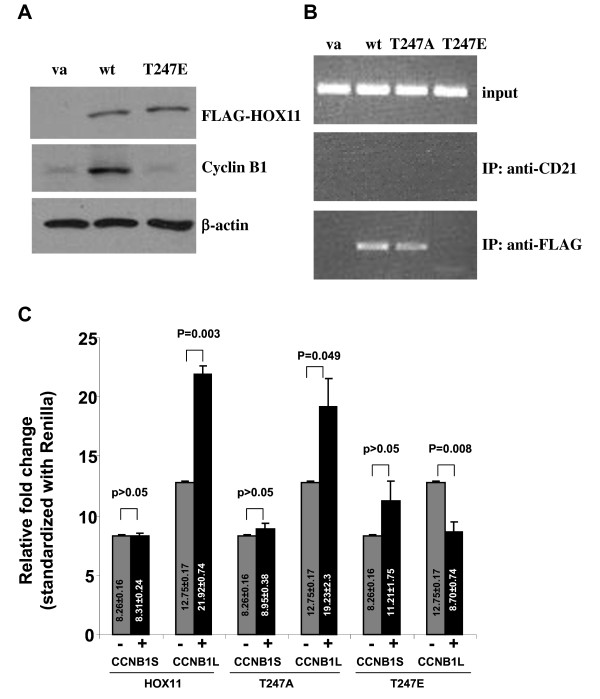
**Phosphorylation of HOX11 Inhibits Transactivation of the Cyclin B1 (*CCNB1) *Promoter**. **(A) **Immunoblot of cyclin B1 levels following transfection of FLAG-tagged HOX11 wildtype and mutant cDNA. Vector alone (va), wildtype HOX11 (wt) and HOX11 Thr247Glu (T247E) are indicated. **(B) **Chromatin immunoprecipitation (ChIP) analysis using an anti-FLAG antibody on the *CCNB1 *promoter region following transfection of FLAG-tagged HOX11 wildtype and mutant cDNA. **(C) **Promoter-reporter assays for activation of the *CCNB1 *promoter containing or lacking the TAAGTG consensus site (CCNB1L-LUC, Long and CCNB1S-LUC, Short, respectively) following transfection of FLAG-tagged HOX11 wildtype and HOX11 T247A and HOX11 T247E mutant proteins. Each bar represents the fold ratio of luciferase activity between HOX11-deficient and HOX11-expressing cultures for each promoter-reporter construct. Each transfection was performed on three independent cultures. The results are representative of three independent experiments. Arbitrary luciferase units were standardized with Renilla.

Next, we investigated the effect on cyclin B1 transactivation of a HOX11 point mutant in which the Thr247 was substituted with a glutamic acid (T247E), a frequently used technique to partially mimic the negative charge introduced by a phosphate group. This approach has been used to simulate a phosphorylation-induced alteration of DNA binding activity of other homeodomain-containing proteins [32,33]. Western immunoblot analysis revealed low-level expression of cyclin B1 in NIH 3T3 cells which was elevated in the presence of HOX11. By contrast, the HOX11 T247E mutant was unable to upregulate cyclin B1 expression (Figure 5A). In accordance, ChIP analyses revealed a decreased association of the HOX11 T247E mutant with the *CCNB1 *promoter whereas the HOX11 T247A mutant showed similar association with the CCNB1 promoter as wild type HOX11 (Figure 5B). EMSA analyses further supported the binding of the HOX11 T247A mutant protein to the TAAGTG target site and no binding by the HOX11 T247E mutant protein to the HOX11 consensus site (Additional file 3, Figure S3). Additional luciferase reporter assays comparing the transactivation of the cyclin B1 promoter containing the TAAGTG HOX11 target sequence (CCNB1L) with the promoter lacking the TAAGTG binding site (CCNB1S) in the presence or absence of HOX11 indicated that the HOX11 protein did not impact transactivation of the CCNB1 S promoter (Figure 5C). In contrast, cells transfected with the CCNB1L construct and HOX11 showed a statistically significant increase in luciferase activity relative to cells lacking the HOX11 protein. To assess the role of phosphorylation of the HOX11 protein in the regulation of the cyclin B1 promoter, cells were cotransfected with either CCNB1 S or CCNB1L in combination with either the HOX11 T247E or HOX11 T247A mutants. Reporter assays demonstrated an inability of the HOX11 T247E mutant to transactivate either the long or short cyclin B1 promoter and, surprisingly, showed significant repression of transcription in the absence of the TAAGTG sequence. The HOX11 T247A mutant was fully functional with respect to transactivation of the *CCNB1 *promoter, indicating that the fidelity of the threonine residue was not the critical factor to HOX11-mediated cyclin B1 upregulation, but rather, the loss of function associated with modification to a glutamic acid residue or following phosphorylation was likely due to a steric interference with DNA binding.

### Phosphorylation of HOX11 in clinically relevant T-cell lines impacts the mitotic spindle checkpoint

**Figure 6:**
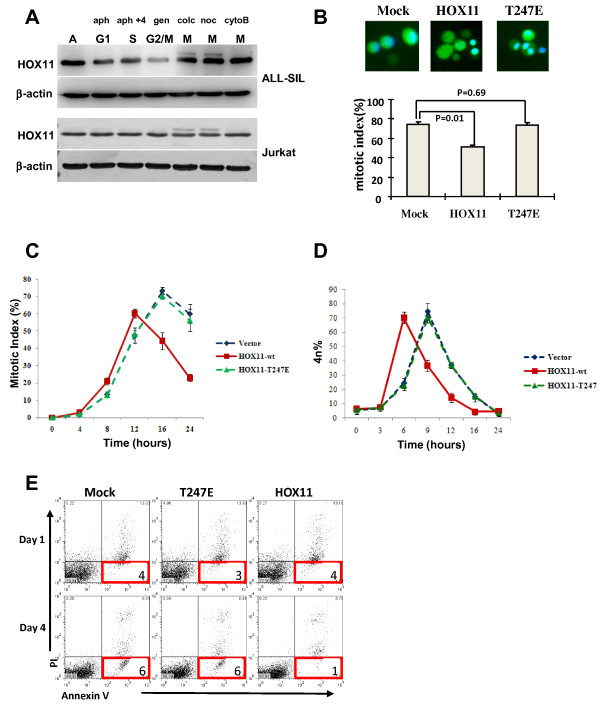
**Phosphorylation of HOX11 During Mitosis in T-Cells is Associated with Aberrant Spindle Checkpoint Arrest and Reduced Early Apoptotic Cells in Cultures Expressing Wild-Type HOX11 but not the HOX11 T247E Mutant**. **(A) **Immunoblot analysis of HOX11 and β-actin in ALL-SIL cells (upper panel) and Jurkat cells transiently transfected with HOX11 (lower panel). Cell lysates were prepared following treatment with aphidocolin (aph) to arrest cells in G1, aphidocolin followed by a 4 h release into fresh medium (aph+4) to arrest cells in S phase, genistein (gen) to arrest cells in G2/M and colchicines (colc), nocodazole (noc) or cytochalasin B (cytoB) to arrest cells in M. (A): asynchronous culture. **(B) **Mitotic index of Jurkat cells transiently transfected with empty vector, wild type HOX11 or the HOX11 T247E mutant then synchronized in mitosis with nocodazole. Nuclei were stained with Hoechst33342 (blue) and mitotic index determined by scoring condensed nuclei. Results are representative of three independent experiments. Error bars indicate the standard deviation. **(C) **Mitotic index of Jurkat cells transfected with wildtype and mutant HOX11. Nocodazole treated cells were harvested at 4-hour intervals and mitotic index determined. **(D) **Dysregulated HOX11 expression accelerates transit through the G2/M phase of the cell cycle. Jurkat cells were transfected with wildtype or mutant vectors. G1/S synchronized cells were released into the cell cycle then harvested at different time points and analyzed by flow cytometry for 4n DNA content by PI-staining. **(E) **One or four days post transfection, Jurkat cells expressing vector alone, HOX11-wt or HOX11-T247E cDNA were stained with Annexin V and PI and analyzed by flow cytometry to assess cell death. Percentages of early apoptotic cells are indicate. The results are representative of three independent experiments.

Given the role of HOX11 in T lymphoid diseases, we sought to determine whether this phenomenon could be recapitulated in a T cell system. Using the T-ALL cell line, ALL-SIL, which carries a t(10;14) translocation concomitant with high levels of HOX11 expression [3] and the TAL1^+ ^Jurkat T-ALL cell line [34] transfected with the HOX11 cDNA, we observed a similar pattern of HOX11 phosphorylation in T cell lines as was seen in HOX11-expressing NIH 3T3 cells with the additional low mobility band only being detected in cells exposed to mitotic arresting agents (Figure 6A). Thus the phosphorylation of HOX11 following nocodazole or colchicine treatment was reproducible in clinically relevant T-cell lines and may be applicable to HOX11 disease pathology.

We previously reported that dysregulated expression of HOX11 contributed to the development of lymphoma by allowing cells with missegregated chromosomes to aberrantly bypass the spindle checkpoint although it remained unclear whether this effect was dependent on the Thr247 residue [28,29]. Therefore, to assess the role of Thr247 phosphorylation in aberrant spindle checkpoint bypass, Jurkat cells transfected with wild-type HOX1l or the T247E mutant were arrested in mitosis with low dose nocodazole and the mitotic index compared 17 hours later by immunofluorescence microscopy by scoring nuclear morphology. Under these conditions, cells expressing wild-type HOX11 exhibited abnormal metaphase arrest with only 51% of cells showing nuclear condensation whereas 73% and 74% of HOX11 T247E or mock transfected cells, respectively, showed nuclear condensation (Figure 6B, Additional file 4, Figure S4). Moreover, time course studies revealed that HOX11-expressing cells showed a premature decline in the mitotic index and improper progression through the spindle checkpoint as compared with vector alone and HOX11 T247E-expressing cells (Figure 6C). By 16 hours, more than 50% of HOX11-expressing cells had escaped the mitotic arrest while 70% of vector control and HOX11 T247E-expressing cells had remained blocked in mitosis. Interestingly, even in the absence of microtubule inhibitors and spindle checkpoint arrest, HOX11-expressing cells showed accelerated progression through mitosis as compared with mock and HOX11 T247E transfected cells (Figure 6D).

Of note, studies assessing cell death by flow cytometry of annexin V and propidium iodide stained cells indicated that Jurkat cells expressing wild-type HOX11 exhibited a reduced frequency of early-stage apoptotic cells relative to mock and HOX11 T247E transfected cells suggesting that HOX11 confers a survival advantage to cells (Figure 6E). Combined, these data are consistent with the hypothesis that HOX11 modulated expression of cyclin B1 via phosphorylation of Thr247 and that cyclin B1, possibly in combination with other HOX11 target genes, plays a role in the dysregulation of the spindle activation checkpoint and transition through the M phase of the cell cycle. Furthermore, dysregulated expression of cyclin B1 may confer a survival advantage to cells with missegregated chromosomes.

## Discussion

The multiple reports of deregulation of cell cycle checkpoints associated with perturbations in *HOX11 *expression [24,25,27-29] prompted us to assess the cell cycle-dependent expression of the HOX11 oncoprotein. In a HOX11-3T3 fibroblast cell line in which the *HOX11 *gene was constitutively active, we observed a post-translational modification of HOX11 which correlated with increased numbers of mitotic cells following treatment with microtubule destabilizing compounds. Radiolabelling studies using several HOX11 truncation and point mutants revealed that this modification was the result of phosphorylation of the HOX11 protein at Thr247 within the homeodomain.

The 60-amino acid homeodomain is a conserved DNA binding protein domain consisting of an N-terminal arm and three α helices that are separated by a loop and a turn. DNA binding of the homeodomain is mediated by the third recognition helix and the N-terminal arm with several conserved amino acids, including Arg3, Arg5, Ile47, Gln50 and Asn51, playing a major role in DNA recognition. Three of the residues, Arg5, Gln50 and Asn51, are conserved in the homeodomains of HOX11 gene family members whereas the two other residues are changed from Arg3 to Lys3 and Ile47 to Thr47. Since both Arg3 and Lys3 have side chains with amine groups this change likely does not affect DNA interactions [20]. In contrast, Thr has both methyl and hydroxyl groups in its side chain while Ile contains only a hydrophobic methyl group and this amino acid change is associated with a change in the DNA recognition sequence from TAATNN for canonical homeodomains to TAAGTG for HOX11 gene family members [20]. Thus the Thr in position 247 in the HOX11 protein plays a critical role in determining the specific DNA base at the fourth position of the binding motif.

The data presented in this report indicated that phosphorylation of HOX11 on Thr247 limited the binding of the HOX11 protein to the promoter region of cyclin B1. Furthermore, HOX11 was phosphorylated only during M phase of the cell cycle resulting in transient inhibition of HOX11 DNA binding activity. Since Thr247 makes specific contact with nucleic acids within the major groove of the DNA double helix, phosphorylation of this amino acid residue places a negative charge close to the phosphates of DNA thereby interfering with DNA binding through electrostatic repulsion. Moreover, replacement of Thr247 with glutamic acid to mimic phosphorylation decreased DNA binding activity. Thus phosphorylation of Thr247 provides a mechanism for cells to inactivate target gene expression as cells enter the M phase and reactivate expression as cells undergo cytokinesis and re-enter the G1 phase of the cell cycle.

Phosphorylation represents a common mechanism in regulating the DNA binding capacity of homeodomain-containing transcription factors including the Drosophilia homeodomain proteins Antennapaedia [35], Engrailed-2 [33,36], Sex combs reduced (SCR) [37] and Even-Skipped [38] and the mammalian homeodomain proteins CDP/Cux [39], hSIX1 [40], the POU transcription factors Oct-1, GHF-1 and Pit-1[41-43], the NK-like homeodomain proteins, Csx/Nkx2.5 [44], and PRH/Hex [45] and the clustered homeobox genes HOXA9 [46], HOXA10 [47-50], HOXB6 [51] and HOXB7 [52]. In some cases, as is the case with HOX11, phosphorylation occurs at conserved sites located within the homeodomain affecting target gene expression and transforming potential. In particular, the *HOXA9 *and *HOXA10 *homeobox genes are frequently over-expressed in acute myeloid leukemias (AMLs). HOXA9 has been reported to be phosphorylated on serine, threonine and tyrosine residues within its homeodomain with phosphorylation correlating with decreased transforming capability in myeloid and murine bone marrow cells [46,53]. Two conserved tyrosine residues in the homeodomain of HOXA10 are phosphorylated during cytokine induced differentiation of myeloid progenitors resulting in decreased HOXA10 binding affinity for regulatory elements of myeloid-specific genes [47-49,54]. The proline-rich homeodomain protein (PRH)/HEX functions early in embryonic vascular and hematopoietic development[55] and as an oncoprotein in T cells [56-58]. Both HOX11 and PRH/HEX are members of the NK-like subclass of homeodomain proteins. Similar to HOX11, PRH has a proline-rich region in the amino terminus of the protein which is involved in its interaction with a variety of proteins including Groucho/TLE and transcription factor eIF-4E (eukaryotic initiation factor 4E) [59-64]. PRH is phosphorylated at two conserved amino acids within the homeodomain with phosphorylation inhibiting DNA binding and transcriptional repression of target genes [45]. In other cases, cell cycle specific phosphorylation of homeodomain proteins effects cell cycle progression [41,42,65-67]. For example, Six1 contributes to the regulation of the G2/M checkpoint with phosphorylation during interphase and mitosis inhibiting DNA binding activity [40]. Moreover, the POU transcription factors GHF-1 and Oct-1 undergo mitosis-specific phosphorylation on amino acids within the homeodomain with phosphorylation correlating with *in vitro *and *in vivo *inhibition of DNA binding activity [41,42,66]. Thus M-phase specific phosphorylation may represent an important mechanism for regulating gene expression as cells progress through the stages of the cell cycle.

We observed that the transcriptional activity of the HOX11 oncoprotein was modulated by phosphorylation of the Thr247 residue. Specifically, the ability of HOX11 to modulate levels of cyclin B1 expression was impaired by a form of HOX11 that contained an acid amino acid substitution at Thr247 that mimicked a constitutively phosphorylated HOX11. Moreover, we observed that the Thr247-dependent upregulation of cyclin B1 was associated with dysfunction of the spindle assembly checkpoint. In this respect, our findings are consistent with previous studies in delineating an uncoupling between genes targeted by the Thr247 residue associated with deregulating the spindle assembly checkpoint and HOX11 target genes involved in cell immortalization [19]. These data, therefore, reaffirm the belief that HOX11 target genes fall into two groups: (1) those regulated *via *Thr247-directed interactions with promoter regions containing the HOX11 consensus recognition sequence TAAGTG, and (2) those regulated through association of promoter regions with HOX11-PBX heterodimers [19].

On the basis of the DNA binding activity of the HOX11 homeodomain and its ability to transactivate reporter gene expression through both TATA-containing and TATA-less promoters [19], it has long been thought that HOX11 functions as a transcriptional regulator. Moreover, several putative HOX11 gene targets, including *Aldh1a1*, *Wt*, *c-kit *and *Vegfc *[21,68-70], have been identified. However, with the exception of its association with its own promoter, the binding of HOX11 to the promoter region of target genes has not been convincingly demonstrated and thus the mechanism of HOX11 homeodomain dependent transcriptional regulation of gene targets remains to be elucidated. The inability to demonstrate target DNA sequence binding has led to speculation that HOX11 regulates gene expression through specific protein-protein interactions with cofactors such as PBX [23], Meis [71] or Groucho/transducin-like Enhancer of split (Gro/TLE) [63] and components of the basal transcriptional machinery, including CTF1 [72]. However, systematic mutational analysis indicated that regulation of *Aldh1a1 *and the oncogenic properties of HOX11 are both dependent on an intact homeodomain [19,22] suggesting that DNA binding is required for HOX11 target gene regulation. The studies described herein help clarify the mechanism of HOX11 mediated transcriptional regulation in that they provide the first evidence of the direct binding of HOX11 to a target TAAGTG sequence located more than 4 kb upstream of the cyclin B1 transcription initiation site. This is consistent with a model in which the homeodomain of HOX11 recognizes and binds to target DNA sequences in the distal promoter region and, once recruited, modulates transcriptional activity by folding or looping back and interacting with the basal transcriptional machinery.

The physiological relevance of HOX11 phosphorylation was demonstrated with respect to its effects on cyclin B1 expression and spindle assembly checkpoint regulation. Expression of wild type HOX11 increased expression of cyclin B1 and this elevated expression was associated with a reduction in transition time through the G2/M checkpoint, a reduced mitotic index and a reduction in the frequency of early apoptotic cells. In contrast, the threonine-to-glutamic acid substitution mutant resulted in a return to normal levels of cyclin B1 expression, cell cycle kinetics, mitotic index and frequency of early apoptotic cells.

Cyclin B1 is the regulatory subunit of cyclin dependent kinase (CDK1). The CDK1/cyclin B1 complex is critical for the control of G2/M transition as it controls the centrosome cycle and the onset of mitosis (reviewed by [73]. The CDK1/cyclin B1 complex phosphorylates numerous target proteins during G2 and early mitosis triggering multiple processes including centrosome separation, nuclear envelope breakdown and chromosome condensation. Transition through metaphase is regulated by the spindle assembly checkpoint which ensures bipolar attachment of all chromosomes to the mitotic spindle. Once all chromosomes are aligned at the metaphase plate, chromosome segregation is mediated by the anaphase promoting complex/cyclosome (APC/C), an E3 ubiquitin ligase responsible for targeting cyclins, including cyclin B1, for degradation. Degradation of cyclin B1 and the associated inactivation of the CDK/cyclin B1 complex allows chromatid separation, chromosome decondensation, reformation of the nuclear envelope and cytokinesis. Given that the degradation of cyclin B1 is critical for entry of cycling cells into anaphase it was surprising that cells expressing the nonphosphorylatable HOX11 T247A mutant and thus unable to downregulate cyclin B1 expression appear to undergo normal mitosis and retain cellular viability. It is possible that other HOX11 target genes involved in the shuttling of cyclin B1 between the nucleus and cytoplasm, its phosphorylation-dependent import and accumulation in the nucleus at prophase or its degradation during the metaphase to anaphase transition may also be regulated in a Thr247 phosphorylation specific manner. Consequently the extended expression of cyclin B1 into anaphase in cells expressing the HOX11 T247A mutant might be countered by these target genes that are no longer regulated at the anaphase transition. In support of this, expression profiling studies undertaken by our group have indicated that Plk1 and components of the APC are deregulated in HOX11transgenic B cells although studies have not yet been initiated to determine whether these HOX11 target genes are also regulated by Thr247 phosphorylation.

Given the essential role of cyclin B1 in the regulation of the G2/M cell cycle checkpoint, it is not surprising that deregulated cyclin B1 expression has been linked to malignant transformation [73]. In normal cells, expression of cyclin B1 begins at S phase with protein accumulation reaching maximal levels at the onset of mitosis. Cyclin B1 is subsequently rapidly degraded at the metaphase-anaphase transition. In primary tumour tissues and leukemia cell lines, accumulation of cyclin B1 is detected in the G1 phase [74-76]. In addition, elevated cyclin B1 has been reported in numerous cancers including gastric [77], colorectal [78], head and neck squamous cell carcinoma [79,80] and non-small-cell lung cancer [81,82]. Deregulated cyclin B1 expression is associated with poor prognosis [78,80,82], histological grade of differentiation and vascular invasion [81]. Elevated cyclin B1 expression often precedes tumour immortalization and aneuploidy suggesting that deregulated expression of cyclin B1 is an early tumorigenic event [83,84]. Since HOX11-expressing malignancies are associated with deregulation of the G2/M cell cycle checkpoint [24-27] and chromosome missegregation [28,29], pharmacological inhibition of cyclin B1 could provide a specific intervention for the treatment of T-ALL.

## Conclusions

Our results demonstrate that the transcriptional activity of HOX11 is regulated by temporal phosphorylation of Thr247 in a cell cycle-specific manner and that this phosphorylation modulates the expression of the target gene, cyclin B1. Since it is likely that Thr247 phosphorylation regulates DNA binding activity to multiple HOX11 target sequences, it is conceivable that phosphorylation functions to regulate the expression of HOX11 target genes involved in the control of the mitotic spindle checkpoint.

## Competing interests

The authors declare that they have no competing interests.

## Authors' contributions

EC conceived of and initiated the project, as well as designed and carried out experiments and drafted the manuscript. XH designed and carried out experiments and contributed to data analysis and writing and editing of the manuscript. YZ performed cell culture and ChIP experiments. Y-JL contributed to EMSA. AC, EY and YB-D analyzed data, provided critical expertise and contributed to the editing of the manuscript. MRH supervised the project and finalized the manuscript. All authors read and approved the final manuscript.

## Supplementary Material

Additional file 1 **Expression of HOX11 in Cell Lines**. Immunoblot analysis of HOX11 and b-actin showing similar levels of HOX11 expression in cell lines. Lane 1: 3T3-vector alone, lane 2: 3T3-HOX11, lane 3: K3P, lane 4: ALL-SIL, lane 5: Jurkat-vector alone, lane 6: Jurkat-HOX11-wt, lane 7 Jurkat-HOX11-T247E, lane 8: Jurkat-HOX11-T247A. Click here for file

Additional file 2 **Titration of staurosporin showing HOX11 phosphorylation over a range of staurosporin concentrations**. HOX11-3T3 cultures were supplemented with varying concentrations of staurosporin ranging from 0 mM to 500 mM in the presence or absence of 500 nM nocodazole. Click here for file

Additional file 3 **EMSA analysis showing binding of the wild type HOX11and HOX11 T247A mutant proteins to the TAAGTG target sequence located ~4.5 kb upstream of the translation start site**. Double-stranded oligonucleotide targets were incubated with nuclear extracts derived from Jurkat cell lines stably expressing an empty flag-vector, flag-HOX11-wt, flag-HOX11-T247 or flag-HOX11-T247E. Reactions included a 23 oligonucleotide target derived from sequences located ~4.5 kb upstream of the cyclin B1 translation start site or a mutant target. Other than the TAAGTG HOX11 binding site, the target sequence does not contain known transcription factor binding sites. EMSA analyses showed binding to the target oligonucleotide sequence by specific factors present in Jurkat cells stably expressing the wild type HOX11 protein and HOX11 T247A mutant protein (left panel, lanes 4-7) but no binding of factors present in Jurkat cells stably expressing the HOX11T247E mutant protein (left panel, lanes 8-9). Mutation of the TAAGTG HOX11 binding site in target oligonucleotides prevented factor binding (right panel, lanes 4-7). The presence of the wild type HOX11 protein and the mutant HOX11T247A protein within the factor complex was confirmed by supershift analysis using an anti-flag antibody. Lane 1: free probe. Lane 2: probe + flag-vector. Lane 3: probe + flag-vector + anti-flag antibody. Lane 4: probe + flag-HOX11-wt. Lane 5: probe + flag-HOX11-wt + anti-flag antibody. Lane 6: probe + flag-HOX11-T247A. Lane 7: probe + flag-HOX11-T247A + anti-flag antibody. Lane 8: probe + flag-HOX11-T247E. Lane 9, probe + flag-HOX11-T247E+ anti-Flag antibody. Click here for file

Additional file 4 **Enhanced viability of Jurkat cells stably expressing HOX11**. Jurkat cells stably expressing an empty flag-vector, flag-HOX11-wt or flag-HOX11-T247E cDNA were subjected to mock Amaxa nucleofection in Solution V using program X-001. Cells were stained with Annexin V and propidium iodide (PI) and analyzed by flow cytometry to assess cell death prior to nucleofection and 2 days post nucleofection. Numbers shown in lower right quadrants indicate percentages of early apoptotic cells. The results are representative of three independent experiments. Click here for file
