# Supplementary figures and images for: Phosphorylation of HOX11/TLX1 on Threonine-247 during mitosis modulates expression of cyclin B1
Source: Mol Cancer. 2010 Sep 16;9:246. doi: 10.1186/1476-4598-9-246 (PMC2949800; doi:10.1186/1476-4598-9-246)

## Slide 1
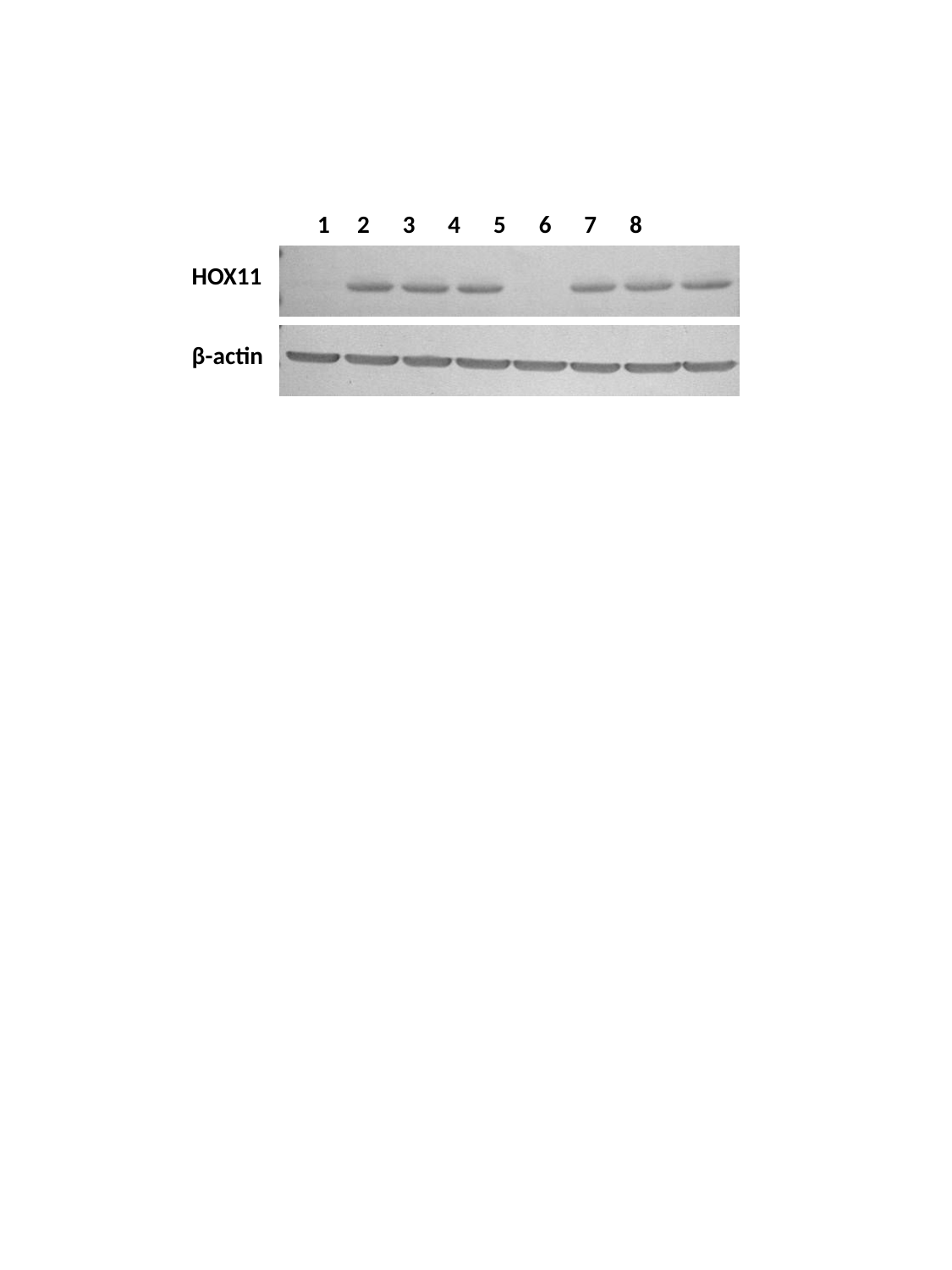

1 2 3 4 5 6 7 8
HOX11
β-actin

Supplement: Additional file 1 — Expression of HOX11 in Cell Lines. Immunoblot analysis of HOX11 and b-actin showing similar levels of HOX11 expression in cell lines. Lane 1: 3T3-vector alone, lane 2: 3T3-HOX11, lane 3: K3P, lane 4: ALL-SIL, lane 5: Jurkat-vector alone, lane 6: Jurkat-HOX11-wt, lane 7 Jurkat-HOX11-T247E, lane 8: Jurkat-HOX11-T247A. [file 1476-4598-9-246-S1.PPT]

## Slide 1
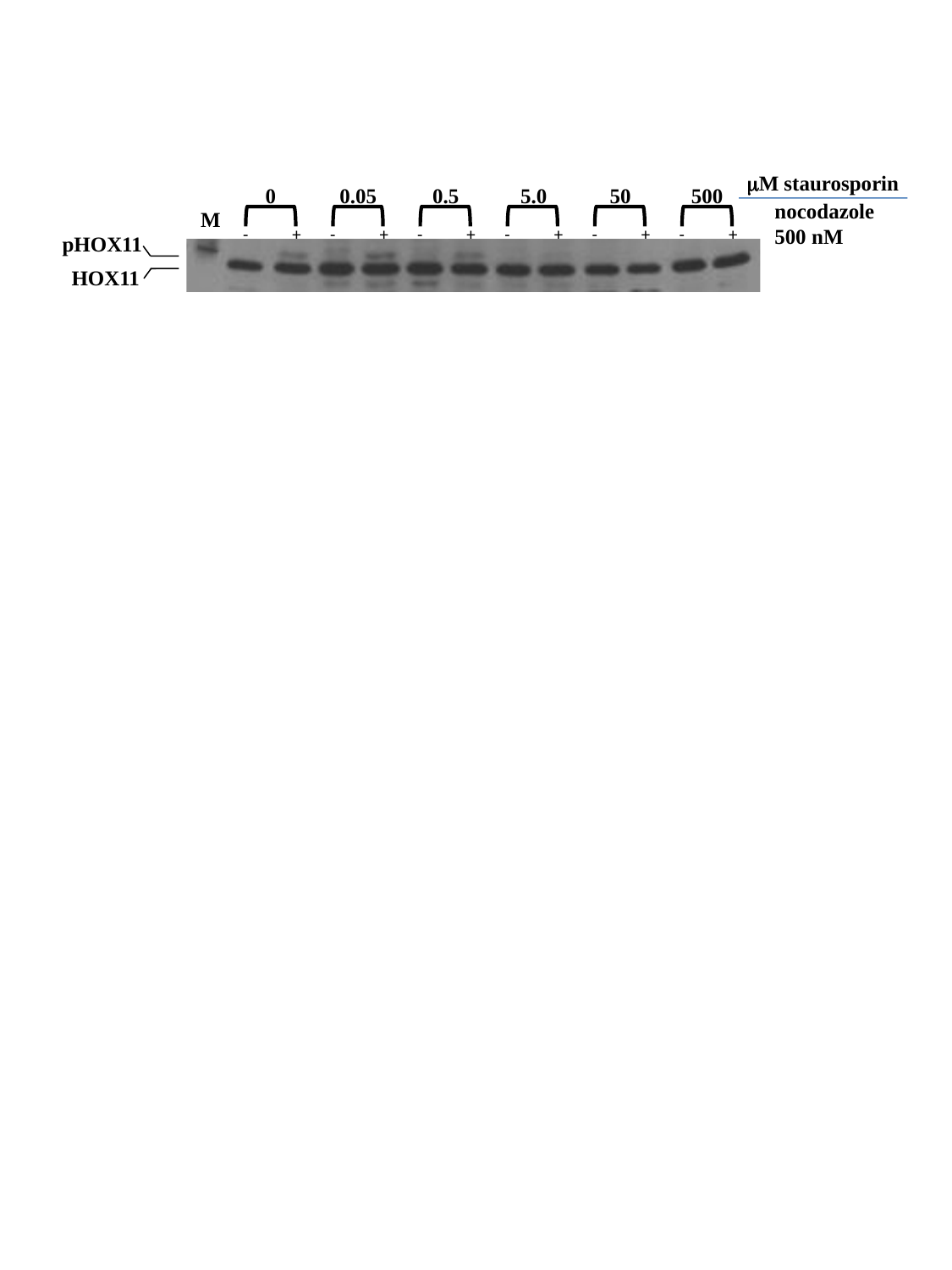

μM staurosporin
0
0.05
0.5
5.0
50
500
nocodazole
500 nM
M
- +
- +
- +
- +
- +
- +
pHOX11
HOX11

Supplement: Additional file 2 — Titration of staurosporin showing HOX11 phosphorylation over a range of staurosporin concentrations. HOX11-3T3 cultures were supplemented with varying concentrations of staurosporin ranging from 0 mM to 500 mM in the presence or absence of 500 nM nocodazole. [file 1476-4598-9-246-S2.PPT]

## Slide 1
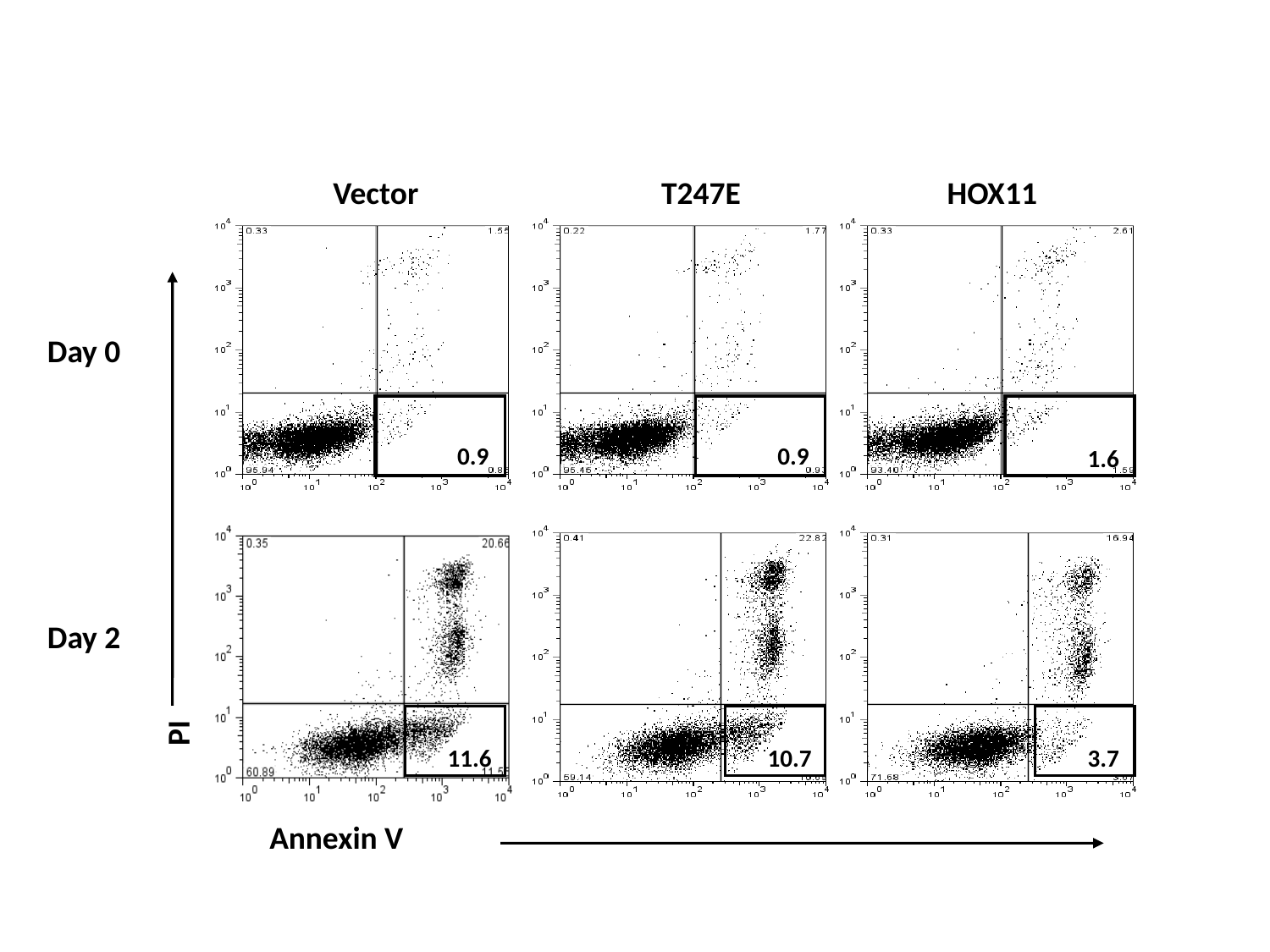

Vector
T247E
HOX11
Day 0
Day 2
PI
Annexin V
0.9
0.9
1.6
11.6
10.7
3.7

Supplement: Additional file 4 — Enhanced viability of Jurkat cells stably expressing HOX11. Jurkat cells stably expressing an empty flag-vector, flag-HOX11-wt or flag-HOX11-T247E cDNA were subjected to mock Amaxa nucleofection in Solution V using program X-001. Cells were stained with Annexin V and propidium iodide (PI) and analyzed by flow cytometry to assess cell death prior to nucleofection and 2 days post nucleofection. Numbers shown in lower right quadrants indicate percentages of early apoptotic cells. The results are representative of three independent experiments. [file 1476-4598-9-246-S4.PPT]
