# Supplementary material for: Phosphorylation of HOX11/TLX1 on Threonine-247 during mitosis modulates expression of cyclin B1
Source: Mol Cancer. 2010 Sep 16;9:246. doi: 10.1186/1476-4598-9-246 (PMC2949800; doi:10.1186/1476-4598-9-246)
Supplement: Additional file 3 — EMSA analysis showing binding of the wild type HOX11and HOX11 T247A mutant proteins to the TAAGTG target sequence located ~4.5 kb upstream of the translation start site. Double-stranded oligonucleotide targets were incubated with nuclear extracts derived from Jurkat cell lines stably expressing an empty flag-vector, flag-HOX11-wt, flag-HOX11-T247 or flag-HOX11-T247E. Reactions included a 23 oligonucleotide target derived from sequences located ~4.5 kb upstream of the cyclin B1 translation start site or a mutant target. Other than the TAAGTG HOX11 binding site, the target sequence does not contain known transcription factor binding sites. EMSA analyses showed binding to the target oligonucleotide sequence by specific factors present in Jurkat cells stably expressing the wild type HOX11 protein and HOX11 T247A mutant protein (left panel, lanes 4-7) but no binding of factors present in Jurkat cells stably expressing the HOX11T247E mutant protein (left panel, lanes 8-9). Mutation of the TAAGTG HOX11 binding site in target oligonucleotides prevented factor binding (right panel, lanes 4-7). The presence of the wild type HOX11 protein and the mutant HOX11T247A protein within the factor complex was confirmed by supershift analysis using an anti-flag antibody. Lane 1: free probe. Lane 2: probe + flag-vector. Lane 3: probe + flag-vector + anti-flag antibody. Lane 4: probe + flag-HOX11-wt. Lane 5: probe + flag-HOX11-wt + anti-flag antibody. Lane 6: probe + flag-HOX11-T247A. Lane 7: probe + flag-HOX11-T247A + anti-flag antibody. Lane 8: probe + flag-HOX11-T247E. Lane 9, probe + flag-HOX11-T247E+ anti-Flag antibody. [file 1476-4598-9-246-S3.PPT]

## Slide 1
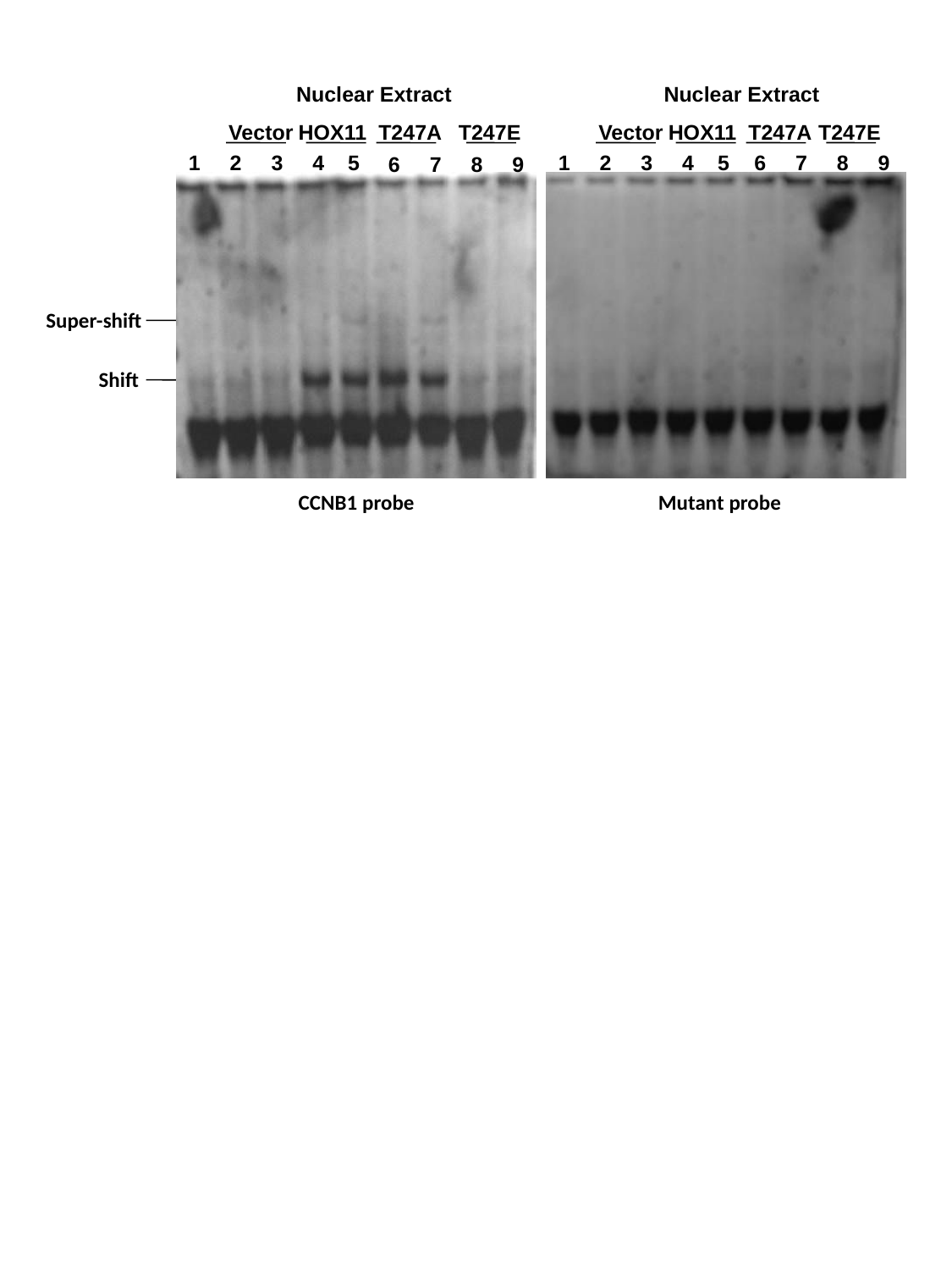

Nuclear Extract
Nuclear Extract
Vector
HOX11
T247A
T247E
Vector
HOX11
T247A
T247E
1 2 3 4 5
1 2 3 4 5
 6 7 8 9
6 7 8 9
Super-shift
Shift
CCNB1 probe
Mutant probe
